# Cytoplasmic β-catenin intensity predicts overall survival in rectal cancer but not colon cancer

**DOI:** 10.3892/ol.2026.15933

**Published:** 2026-10-30

**Authors:** Myoung Hee Kang, Se-Il Go, Dong Chul Kim, Dae Hyun Song, Young-Tae Ju, Won Sup Lee

**Affiliations:** 1 Department of Internal Medicine, Institute of Medical Science, Gyeongsang National University Changwon Hospital, Gyeongsang National University College of Medicine, Changwon, Gyeongsangnam-do 51472, Republic of Korea; 2 Department of Pathology, Institute of Medical Science, Gyeongsang National University Hospital, Gyeongsang National University College of Medicine, Jinju, Gyeongsangnam-do 52727, Republic of Korea; 3 Department of Pathology, Institute of Medical Science, Gyeongsang National University Changwon Hospital, Gyeongsang National University College of Medicine, Changwon, Gyeongsangnam-do 51472, Republic of Korea; 4 Department of Surgery, Institute of Medical Science, Gyeongsang National University Hospital, Gyeongsang National University College of Medicine, Jinju, Gyeongsangnam-do 52727, Republic of Korea; 5 Department of Internal Medicine, Institute of Medical Science, Gyeongsang National University Hospital, Gyeongsang National University College of Medicine, Jinju, Gyeongsangnam-do 52727, Republic of Korea

**Keywords:** β-catenin, rectal cancer, colon cancer, prognosis, immunohistochemistry

## Abstract

Colorectal cancer (CRC) is a common malignancy with a rising global incidence. While genetic alterations such as mutations in APC, KRAS and TP53 are well-established in CRC pathogenesis, the role of epithelial-mesenchymal transition (EMT) and cell adhesion molecules in tumor progression has gained increasing attention. Among key regulators, p53 and β-catenin serve critical roles in EMT and tumor behavior. In the present study, 171 patients with CRC who underwent curative resection between 2000 and 2007 were retrospectively analyzed. Immunohistochemistry was performed to evaluate p53 expression and β-catenin localization (nuclear vs. cytoplasmic). β-catenin staining intensity was classified as negative, weakly positive or strongly positive. Overall survival (OS) was assessed using Kaplan-Meier analysis and the log-rank test, and independent prognostic factors were identified using multivariate Cox proportional hazards regression analysis. Neither p53 expression nor nuclear β-catenin intensity was significantly associated with OS in either colon or rectal cancer. By contrast, among patients with rectal cancer (n=103), those with lower cytoplasmic β-catenin intensity exhibited significantly worse OS compared with those with higher intensity (median OS, 94 months vs. not reached; P=0.026). This association remained significant in multivariate analysis (hazard ratio, 2.27; 95% CI, 1.06–4.85; P=0.034), independent of TNM stage and other covariates. These findings indicated that β-catenin intensity may serve as a clinically relevant prognostic biomarker in rectal cancer, whereas p53 expression and nuclear β-catenin intensity have limited prognostic value.

## Introduction

Colorectal cancer is the fourth most commonly diagnosed malignancy in the United States (1) and continues to show a steadily increasing incidence in Korea (2). The molecular pathogenesis of CRC has been extensively characterized, with genetic alterations involving tumor suppressor genes such as APC, KRAS, TP53, and DCC playing pivotal roles in tumor initiation and progression (3). Beyond genetic mutations, alterations in epithelial cell–cell adhesion structures are critically involved in tumor invasion and metastasis. In particular, epithelial–mesenchymal transition (EMT), a process whereby epithelial cells acquire mesenchymal characteristics and enhanced migratory capacity, is considered a key step in malignant progression (Fig. 1) (4,5).

Multiple transcriptional regulators and adhesion molecules participate in EMT, among which p53 and β-catenin have been widely studied. p53, a tumor suppressor protein encoded by the TP53 gene on chromosome 17, regulates DNA damage repair and apoptosis (6–8). Aberrant p53 expression or functional loss is observed in approximately 40–50% of CRC cases. Emerging evidence suggests that p53 also modulates EMT through pathways such as miR-34, suppressing EMT-related transcription factors including Snail1 and indirectly maintaining E-cadherin-mediated cell adhesion (9,10).

β-catenin was originally identified as a component of adherens junctions, mediating intercellular adhesion through interaction with E-cadherin at the cell membrane. It is now recognized as a central regulator of the Wnt signaling pathway (11). In normal epithelial cells, β-catenin is predominantly localized to the membrane and cytoplasm; however, upon Wnt pathway activation or oncogenic mutation, β-catenin accumulates in the nucleus, where it functions as a transcriptional co-activator of oncogenic target genes (12). Aberrant β-catenin activation can induce EMT and promote tumor invasion and metastasis, and altered β-catenin expression or subcellular localization has been associated with prognosis in various malignancies (13–15).

Previous studies have reported that reduced cytoplasmic β-catenin expression is associated with poorer survival in patients with gastrointestinal cancers, whereas the prognostic significance of nuclear β-catenin expression and p53 status remains inconsistent (16). These findings suggest that the prognostic implications of β-catenin may differ according to its subcellular localization and tumor site (17–20). Therefore, the present study aimed to evaluate the prognostic significance of p53 expression and β-catenin intensity, with particular emphasis on subcellular localization, in patients with colon and rectal cancer.

## Materials and methods

### Patient population

Tumor specimens from patients who underwent curative resection for colorectal cancer at Gyeongsang National University Hospital between 2000 and 2007 were retrospectively reviewed. Among these, 171 patients with sufficient clinical data and available tumor tissue were included in the analysis. The patients ranged in age from 23 to 90 years (median, 64 years). This study was approved by the Institutional Review Board of Gyeongsang National University Changwon Hospital (approval number: 2025-06-011).

### Immunohistochemical analysis of p53 and β-catenin

Immunohistochemistry (IHC) was performed on formalin-fixed, paraffin-embedded tissue sections. For detection of β-catenin, a primary antibody from Boster (PA1212-1) was used at a dilution of 1:100 (0.5 µg/ml). For p53 detection, an antibody from Abcam (ab32049) was used at a dilution of 1:50 (0.5 µg/ml). Heat-mediated antigen retrieval using citrate buffer (pH 6.0) was performed prior to immunostaining. Appropriate positive and negative controls were included in each staining run in accordance with the institutional pathology laboratory's routine quality-control procedures. Archival formalin-fixed, paraffin-embedded breast tissue routinely maintained by the Department of Pathology was used as a negative control for β-catenin immunohistochemistry. This control tissue was not obtained specifically for the present study or from patients in the study cohort.

p53 positivity was defined as moderate to strong nuclear staining in ≥10% of tumor cells. β-catenin expression was evaluated separately in the nucleus and cytoplasm. Nuclear β-catenin expression was assessed semiquantitatively by two experienced pathologists based solely on the intensity of nuclear staining and was scored as 0 (absent), 1+ (weak; faint brown staining), 2+ (moderate; distinct brown staining), or 3+ (strong; intense dark brown staining). The proportion of positively stained tumor cells was not considered in the nuclear scoring system. Cytoplasmic β-catenin staining was classified as negative (weak staining in <5% of cells), weak positive (moderate staining in 5–29% of cells), or strong positive (strong staining in ≥30% of cells) (Fig. S1). The immunohistochemical scoring system used in this study was adapted from established protocols in gastrointestinal oncology to ensure reproducibility. To assess interobserver agreement, IHC slides were independently evaluated by two blinded pathologists. The Cohen's kappa coefficient (κ) was 0.82 (95% CI: 0.75–0.89), indicating excellent interobserver agreement. Discordant cases were jointly reviewed using a multi-head microscope to reach a final consensus.

### Statistical analysis

All statistical analyses were performed using STATA version 18.0 (StataCorp LLC, College Station, TX, USA). Categorical variables were compared using the chi-square test or Fisher's exact test, and continuous variables were analyzed using the Mann-Whitney U test. The association between nuclear and cytoplasmic β-catenin intensity in the rectal cancer cohort was assessed using Fisher's exact test. Overall survival (OS), defined as the interval from diagnosis to death or last follow-up, was selected as the primary study endpoint because the cause of death was unavailable, precluding disease-specific survival (DSS) analysis, and recurrence data were incomplete for reliable recurrence-free survival (RFS) analysis. Survival curves were generated using the Kaplan-Meier method and compared using the log-rank test. Multivariate Cox proportional hazards regression analysis was conducted to identify independent prognostic factors, including sex, age, histological differentiation, TNM stage, p53 expression, and β-catenin intensity. Hazard ratios (HRs) and 95% confidence intervals (CIs) were calculated. A two-sided P-value <0.05 was considered statistically significant.

## Results

### Univariate survival analysis of biomarkers

Immunohistochemical analysis of p53 expression, nuclear β-catenin intensity, and cytoplasmic β-catenin intensity was performed in all 171 patients (Table I). The median follow-up duration for the entire cohort was 133 months.

In patients with colon cancer, neither p53 expression nor nuclear β-catenin intensity was significantly associated with OS (P=0.891 and P=0.683, respectively). Similar results were observed in rectal cancer patients (P=0.755 and P=0.448, respectively; Figs. 2, and 3A and B). Cytoplasmic β-catenin intensity (weak positive vs. strong positive) was not associated with OS in colon cancer (P=0.718; Fig. 3C). However, among rectal cancer patients (n=103), those with lower cytoplasmic β-catenin intensity (weak positive) exhibited significantly shorter OS compared with those with higher intensity (strong positive) (median OS: 94 months vs. not reached; P=0.026; Fig. 3D). Subsequent analyses therefore focused on cytoplasmic β-catenin intensity in the rectal cancer cohort.

### Baseline characteristics according to cytoplasmic β-catenin intensity in rectal cancer

Among the 103 rectal cancer patients, 18 (17.5%) had cytoplasmic β-catenin intensity of weak positive and 85 (82.5%) showed strong positive intensity. Baseline clinicopathological characteristics, including sex, age, histological differentiation, TNM stage distribution, and baseline carcinoembryonic antigen (CEA) levels, did not differ significantly between the two groups (Table II), indicating well-balanced baseline characteristics.

### Multivariate prognostic analysis in rectal cancer

Multivariate Cox proportional hazards regression analysis demonstrated that lower cytoplasmic β-catenin intensity (weak positive) was an independent predictor of worse OS compared with higher intensity (strong positive) (HR 2.271; 95% CI 1.063–4.852; P=0.034), after adjustment for other covariates (Table III). As expected, advanced TNM stage (III/IV) was strongly associated with poor OS (HR 17.856; 95% CI 4.895–65.140; P<0.001). In contrast, p53 expression was not independently associated with OS. A significant association was observed between cytoplasmic and nuclear β-catenin intensity (Fisher's exact test; P<0.001; Fig. 4). However, despite this association, only cytoplasmic β-catenin intensity remained independently associated with overall survival in rectal cancer, whereas nuclear β-catenin intensity showed no prognostic significance.

## Discussion

The primary objective of this study was to evaluate the prognostic significance of p53 expression and β-catenin intensity, considering both nuclear and cytoplasmic localization, in patients with colorectal cancer. Our findings demonstrate that neither p53 expression nor nuclear β-catenin intensity is significantly associated with OS in colorectal cancer. In contrast, lower cytoplasmic β-catenin intensity is independently associated with worse OS in rectal cancer, even after adjustment for established prognostic factors such as TNM stage. As expected, advanced TNM stage (III/IV) was associated with the highest hazard ratio (HR=17.856), reflecting its established role in clinical oncology. Nevertheless, the independent prognostic value of cytoplasmic β-catenin intensity (HR=2.271, P<0.05) remains clinically relevant. Multivariate Cox regression confirmed that low cytoplasmic β-catenin expression serves as an independent prognostic factor, indicating that it provides incremental risk stratification beyond TNM staging. This finding may help identify patients at higher risk of poor outcomes who could benefit from closer postoperative surveillance.

Previous studies have reported heterogeneous results regarding the prognostic role of cytoplasmic β-catenin in gastrointestinal adenocarcinomas (21). Some studies have shown that increased cytoplasmic β-catenin expression is associated with improved survival (16). However, meta-analyses have demonstrated no significant association between cytoplasmic β-catenin expression and overall survival (22). The prognostic relevance of membranous β-catenin localization has also been emphasized, with several studies reporting that reduced membrane β-catenin expression is associated with poorer survival outcomes (15). Furthermore, the progressive redistribution of β-catenin from the cell membrane to the nucleus during the transition from normal colonic epithelium to benign, premalignant, and malignant lesions suggests that its subcellular location is closely related to the biological alterations underlying cancer progression (19).

β-catenin is a multifunctional protein that plays critical roles in both cellular architecture and signal transduction. It serves as a key component of adherens junctions, linking cadherins to the actin cytoskeleton and thereby maintaining cell-to-cell adhesion (18). Simultaneously, β-catenin functions as a transcriptional coactivator in the Wnt signaling pathway (18). In the absence of Wnt signaling, cytoplasmic β-catenin is targeted for degradation by the destruction complex. Upon binding of Wnt ligands to their receptors, this complex is inhibited, leading to cytoplasmic accumulation of β-catenin (16). The accumulated β-catenin subsequently translocates to the nucleus, where it interacts with TCF/LEF transcription factors and activates the expression of genes involved in cell proliferation, differentiation, and survival (18). This process is generally recognized as a key driver of Wnt pathway activation and tumorigenesis (22). Therefore, the present finding that lower cytoplasmic β-catenin intensity is associated with poorer prognosis suggests that the underlying biological mechanisms may be more complex than previously understood.

One possible explanation for these findings is that, in rectal cancer, a certain threshold level of cytoplasmic β-catenin may be necessary to maintain adequate intercellular adhesion through its interaction with E-cadherin at the cell membrane (16). Reduced cytoplasmic β-catenin levels may lead to decreased membrane-associated β-catenin, resulting in weakened cell-to-cell adhesion, enhanced cellular motility, and an increased propensity for invasion and metastasis, ultimately contributing to poorer survival outcomes. Alternatively, low cytoplasmic β-catenin intensity in rectal cancer may reflect a distinct pattern of Wnt signaling dysregulation. Higher cytoplasmic β-catenin levels may be associated with a relatively less invasive phenotype, whereas the reduced levels observed in the weak-positive group may impair cellular functions essential for maintaining epithelial integrity and survival (23). Furthermore, β-catenin is known to interact with multiple other signaling pathways, including EGFR, Hippo, and PI3K/Akt, and the crosstalk among these pathways may differ between colon and rectal cancers. The molecular mechanisms linking low cytoplasmic β-catenin expression to poor prognosis in rectal cancer remain to be fully elucidated, but several pathways may explain this phenomenon. Cytoplasmic depletion of β-catenin may reflect accelerated degradation via the destruction complex or enhanced nuclear translocation, thereby promoting EMT and tumor invasiveness. In addition, disruption of the E-cadherin/β-catenin complex may weaken cell-cell adhesion and contribute to metastatic potential. Although cytoplasmic and nuclear β-catenin expression are biologically related, only cytoplasmic β-catenin intensity was independently associated with prognosis in our cohort. This finding suggests that cytoplasmic β-catenin expression may have greater clinical relevance than nuclear localization for risk stratification in rectal cancer.

Nuclear accumulation of β-catenin is generally regarded as a surrogate marker of Wnt pathway activation and has traditionally been considered a poor prognostic factor (22–25). However, previous studies have reported inconsistent prognostic associations. Consistent with these observations, nuclear β-catenin intensity was not independently associated with overall survival in our cohort, suggesting that nuclear localization alone may not adequately reflect the biologically relevant activity of Wnt pathway in rectal cancer.

p53 is a well-established tumor suppressor gene, and aberrant expression is frequently observed in colorectal cancer. However, the prognostic significance of p53 remains controversial (26). Some studies have reported that p53 expression is associated with poorer survival outcomes (17), whereas others have demonstrated no significant association or even a favorable prognostic impact (26). These discrepancies suggest that the clinical relevance of p53 may depend on the specific pattern of alteration, including p21 status, protein overexpression, or complete loss of expression (27). In the present study, the lack of a significant association between p53 expression and prognosis may be attributable to the heterogeneity of TP53 mutations within this cohort, or to attenuation of any early prognostic effect over a prolonged follow-up period.

This study has several limitations. First, as a retrospective analysis, it is inherently limited in establishing causal relationships, and the single-center design restricts the generalizability of the findings. Second, a notable limitation is the relatively small sample size of the rectal cancer subgroup with weak cytoplasmic β-catenin expression (n=18). This small sample size inherently broadens the confidence intervals, which demands caution when generalizing the statistical robustness of our survival analysis. Despite this limitation, the survival deficit observed in this subgroup was statistically significant; nevertheless, future large-scale, multi-center prospective studies are warranted to narrow the confidence intervals and validate these prognostic trends. Third, the contemporary applicability of these results may be constrained, as patient enrollment (2000–2007) preceded the establishment of current standard treatment strategies. We acknowledge that our study cohort may not fully reflect recent advancements in rectal cancer management. Over the past two decades, the widespread implementation of total mesorectal excision (TME), standardized neoadjuvant chemoradiotherapy (CRT), and more recently, total neoadjuvant therapy (TNT) have significantly improved local control and overall survival rates. These therapeutic shifts could potentially alter the prognostic impact of cytoplasmic β-catenin expression observed in our historical cohort. However, our findings remain highly relevant as they identify a distinct biological subgroup that may exhibit differential treatment responses, providing a baseline for future validation in modern cohorts. Finally, although immunohistochemistry (IHC) is a widely utilized method, it has inherent limitations in quantitatively and functionally reflecting protein activity.

In conclusion, lower cytoplasmic β-catenin intensity is independently associated with poor overall survival in patients with rectal cancer, whereas p53 expression and nuclear β-catenin intensity are not prognostically significant. These findings highlight the importance of β-catenin subcellular localization in rectal cancer prognosis and warrant further validation in larger, prospective studies.

## Supplementary Material

Supporting Data

## Figures and Tables

**Figure 1.**
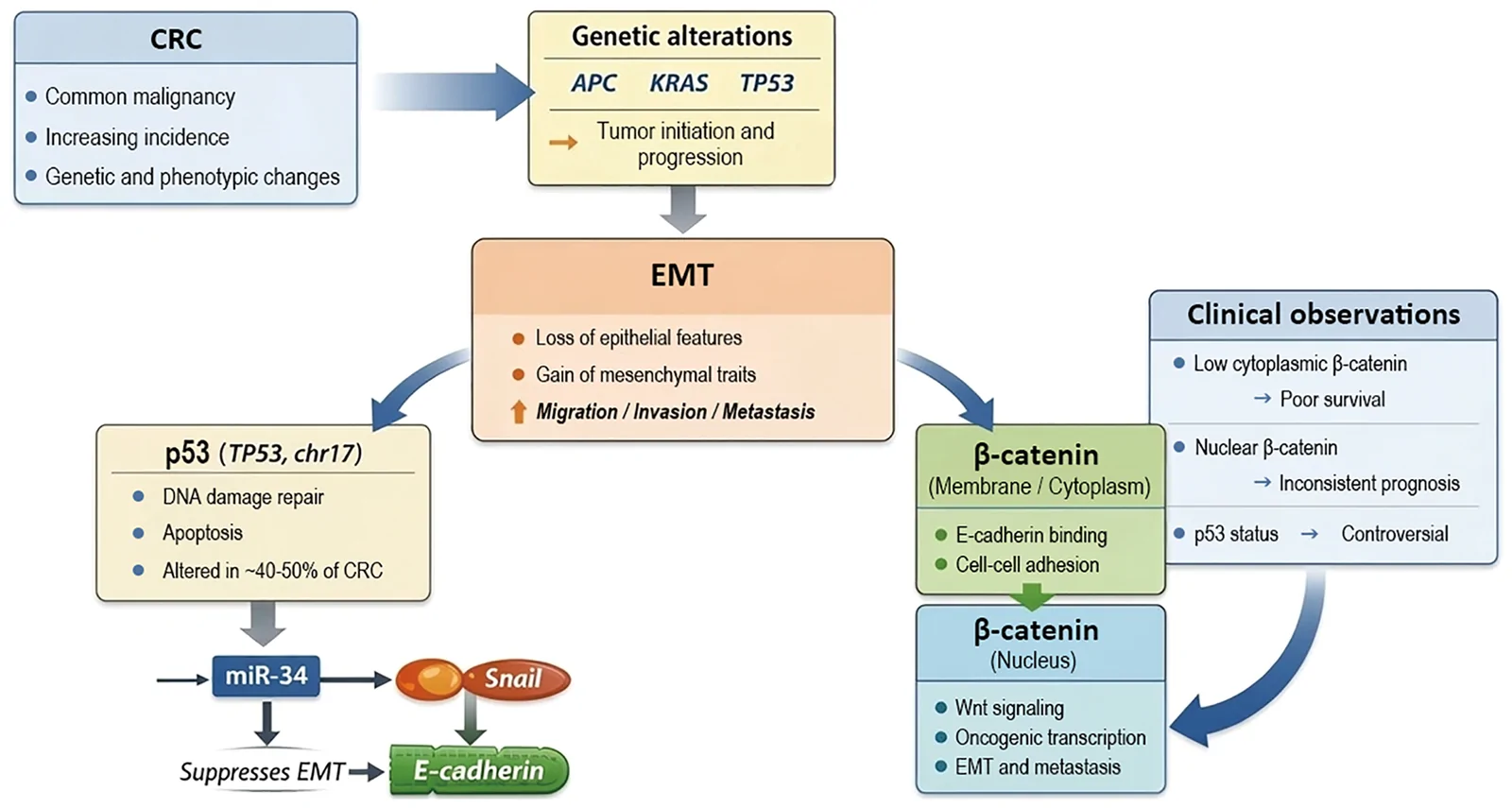
Schematic overview of the mechanisms linking colorectal cancer progression, EMT, p53 and β-catenin. CRC progression is driven by genetic alterations, including APC, KRAS and TP53 mutations, which contribute to tumor initiation and progression. EMT is characterized by the loss of epithelial features and the acquisition of mesenchymal traits, promoting tumor invasion and metastasis. p53 suppresses EMT via the miR-34 pathway through the regulation of Snail and E-cadherin. β-catenin exhibits distinct functional roles depending on its subcellular localization, contributing to cell-cell adhesion at the membrane and in the cytoplasm, while acting as a transcriptional co-activator in the nucleus via Wnt signaling. These mechanisms provide the basis for investigating the prognostic significance of p53 expression and β-catenin localization in colon and rectal cancer. APC, adenomatous polyposis coli; chr, chromosome; CRC, colorectal cancer; EMT, epithelial-mesenchymal transition; KRAS, Kirsten rat sarcoma viral oncogene homolog; miR, microRNA; TP53, tumor protein p53.

**Figure 2.**
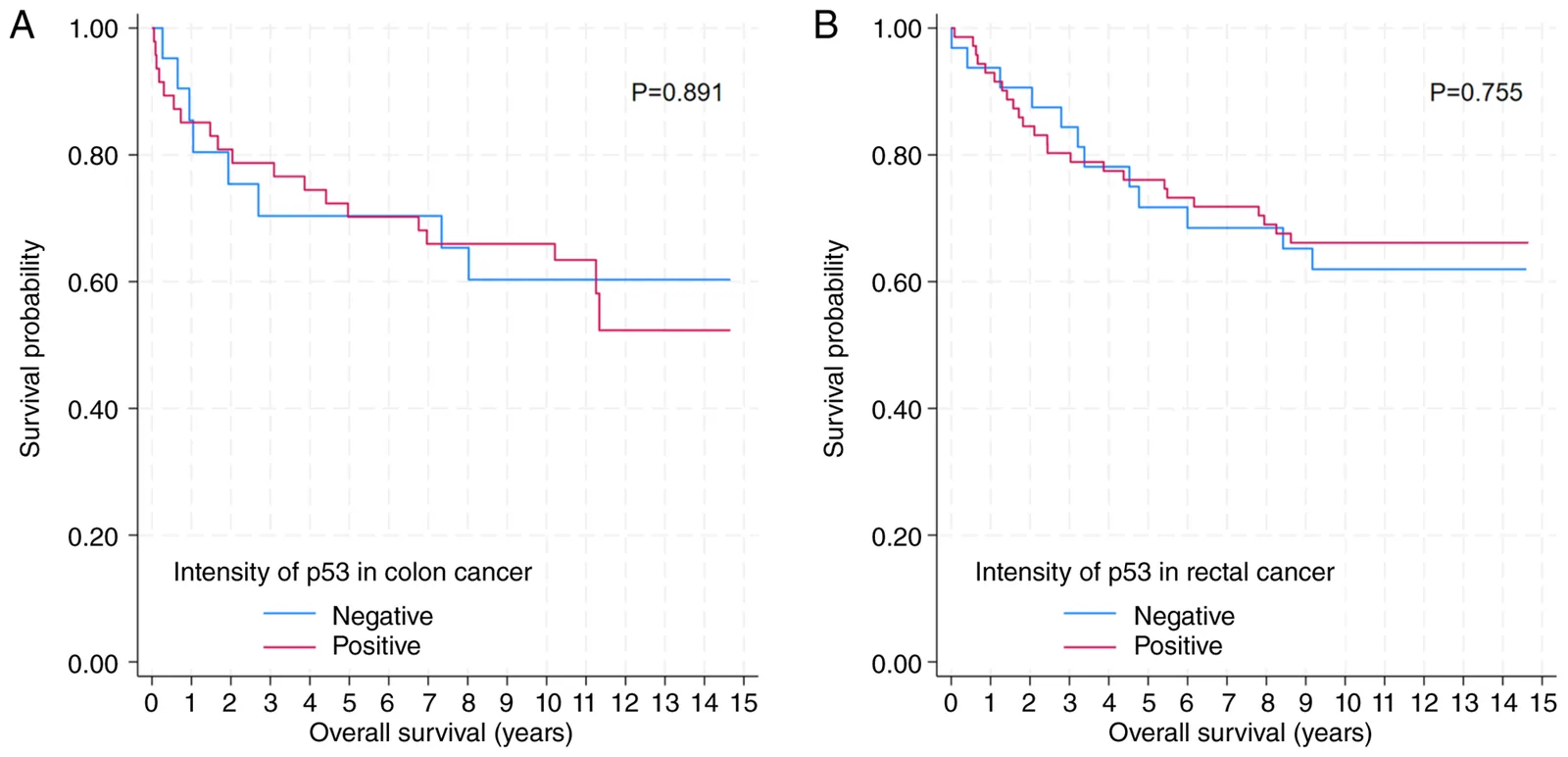
Overall survival according to p53 expression in colon and rectal cancer. (A) Kaplan-Meier overall survival curves in patients with colon cancer stratified by p53 expression status (negative vs. positive). p53 expression was not significantly associated with overall survival in colon cancer (P=0.891; log-rank test). (B) Kaplan-Meier overall survival curves in patients with rectal cancer according to p53 expression status. Similarly, no significant difference in overall survival was observed between p53-negative and p53-positive groups (P=0.755; log-rank test).

**Figure 3.**
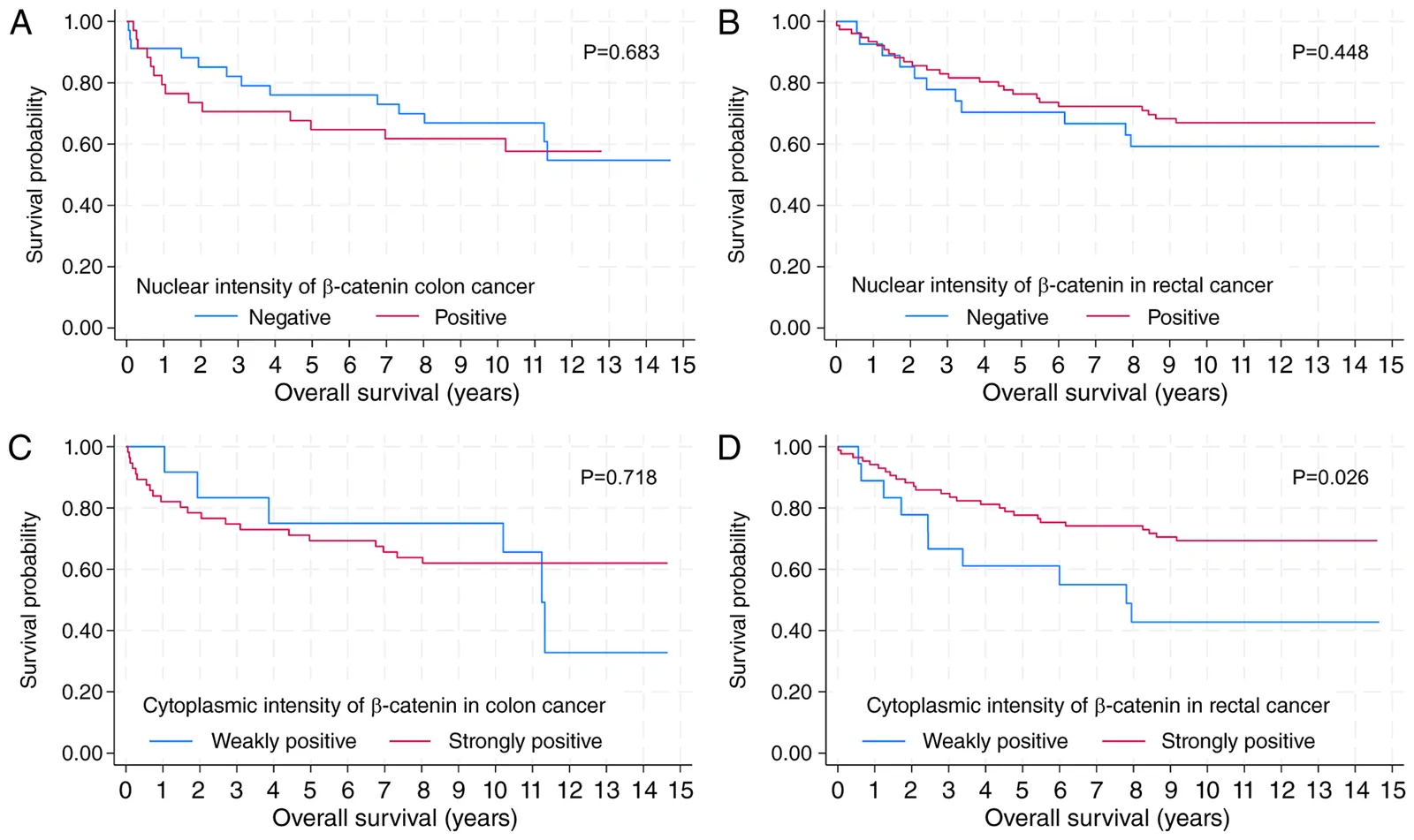
Overall survival according to β-catenin expression in colon and rectal cancer. (A) Kaplan-Meier overall survival curves in patients with colon cancer stratified by nuclear β-catenin expression (negative vs. positive). Nuclear β-catenin expression was not significantly associated with overall survival in colon cancer (P=0.683; log-rank test). (B) Kaplan-Meier overall survival curves in patients with rectal cancer according to nuclear β-catenin expression. No significant difference in overall survival was observed between nuclear β-catenin-negative and -positive groups (P=0.448; log-rank test). (C) Kaplan-Meier overall survival curves in patients with colon cancer stratified by cytoplasmic β-catenin intensity (weakly positive vs. strongly positive). Cytoplasmic β-catenin intensity was not significantly associated with overall survival in colon cancer (P=0.718; log-rank test). (D) Kaplan-Meier overall survival curves in patients with rectal cancer according to cytoplasmic β-catenin intensity. Patients with lower cytoplasmic β-catenin intensity (weakly positive) exhibited significantly worse overall survival compared with those with higher intensity (strongly positive) (P=0.026; log-rank test).

**Figure 4.**
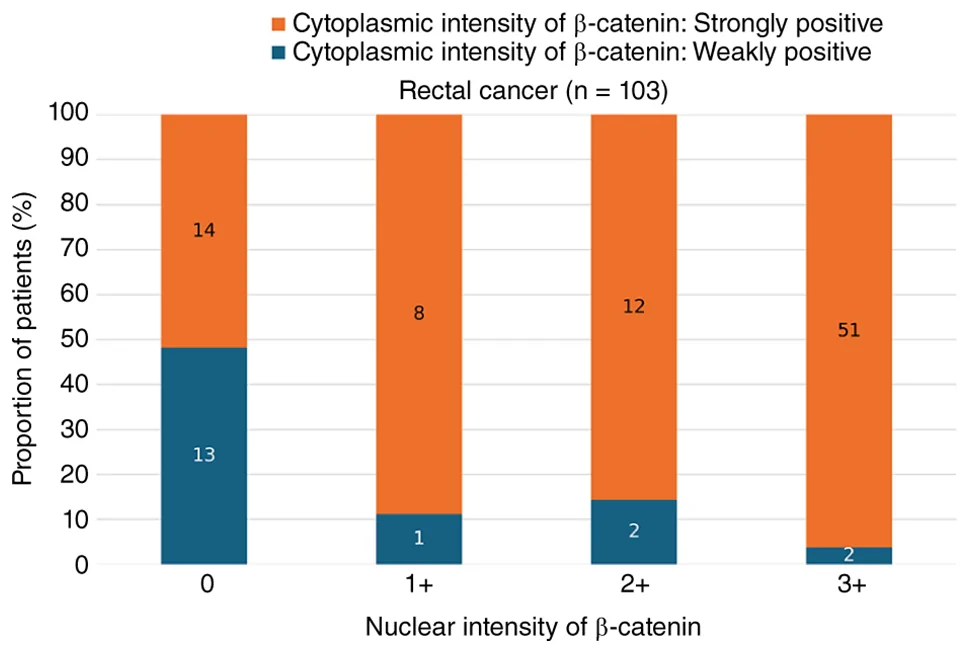
Distribution of weak and strong cytoplasmic β-catenin intensity according to nuclear β-catenin intensity in rectal cancer. Numbers within the bars indicate the number of cases. The association between nuclear and cytoplasmic β-catenin intensity was analyzed using Fisher's exact test (P<0.001).

**Table I.** Immunohistochemical expression of p53 and β-catenin in colon and rectal cancer.

							Cytoplasmic β-catenin	
	p53		Nuclear β-catenin					
							Weakly positive	Strongly positive
Location	Negative	Positive	0	1+	2+	3+		
Colon, n	21	47	34	6	9	19	12	56
Rectal, n	32	71	27	9	14	53	18	85
Total, n	53	118	61	15	23	72	30	141

**Table II.** Baseline characteristics of patients with rectal cancer.

	Cytoplasmic β-catenin intensity		
		
Characteristics	Weakly positive	Strongly positive	P-value
No. (%)	18 (17.5)	85 (82.5)	
Sex, n (%)			0.186
Male	13 (72.2)	47 (55.3)	
Female	5 (27.8)	38 (44.7)	
Median age, years (IQR)	62 (54–70)	63 (58–70)	0.487
Histological			0.394
differentiation, n (%)			
W/D	5 (27.8)	27 (31.8)	
M/D	11 (61.1)	55 (64.7)	
P/D and others	2 (11.1)	3 (3.5)	
TNM stage, n (%)			0.229
I	1 (5.6)	22 (25.9)	
II	7 (38.9)	33 (38.8)	
III	9 (50.0)	27 (31.8)	
IV	1 (5.6)	3 (3.5)	
Mean CEA, ng/ml (SD)	3.9 (8.0)	4.4 (8.2)	0.137

IQR, interquartile range; W/D, well-differentiated; M/D, moderately differentiated; P/D, poorly differentiated.

**Table III.** Multivariate Cox regression analysis of overall survival in rectal cancer.

Variable	HR (95% CI)	P-value
Sex		
Male	Ref.	
Female	0.625 (0.289–1.354)	0.234
Age, years (per year)	1.016 (0.982–1.051)	0.359
Histological differentiation		
W/D	Ref.	
M/D	0.940 (0.423–2.085)	0.878
P/D or others	3.071 (0.828–11.395)	0.094
TNM stage		
I	Ref.	
II	1.685 (0.762–3.725)	0.197
III/IV	17.856 (4.895–65.140)	<0.001
p53		
Negative	Ref.	
Positive	1.103 (0.509–2.393)	0.804
Cytoplasmic β-catenin intensity		
Strong positive	Ref.	
Weak positive	2.271 (1.063–4.852)	0.034

W/D, well-differentiated; M/D, moderately differentiated; P/D, poorly differentiated; HR, hazard ratio; Ref., reference.

## Data Availability

The data generated in the present study are not publicly available due to patient privacy considerations but may be requested from the corresponding author.
