# Supplementary material for: Cytoplasmic β-catenin intensity predicts overall survival in rectal cancer but not colon cancer
Source: Oncol Lett. 2026 Oct 30;32(6):578. doi: 10.3892/ol.2026.15933 (PMC13760667; doi:10.3892/ol.2026.15933)

Figure S1. Representative immunohistochemical staining of β-catenin and negative staining control. (A) Representative rectal cancer tissue exhibiting both weakly positive and strongly positive cytoplasmic β-catenin staining within the same tissue section. (B) Representative image of weakly positive cytoplasmic β-catenin staining in rectal cancer tissue. (C) Representative image of strongly positive cytoplasmic β-catenin staining in rectal cancer tissue. (D) Negative staining control using archival formalin-fixed, paraffin-embedded breast tissue routinely employed by the Department of Pathology for immunohistochemical quality control. Original magnification, x200; scale bar, 100 μm.

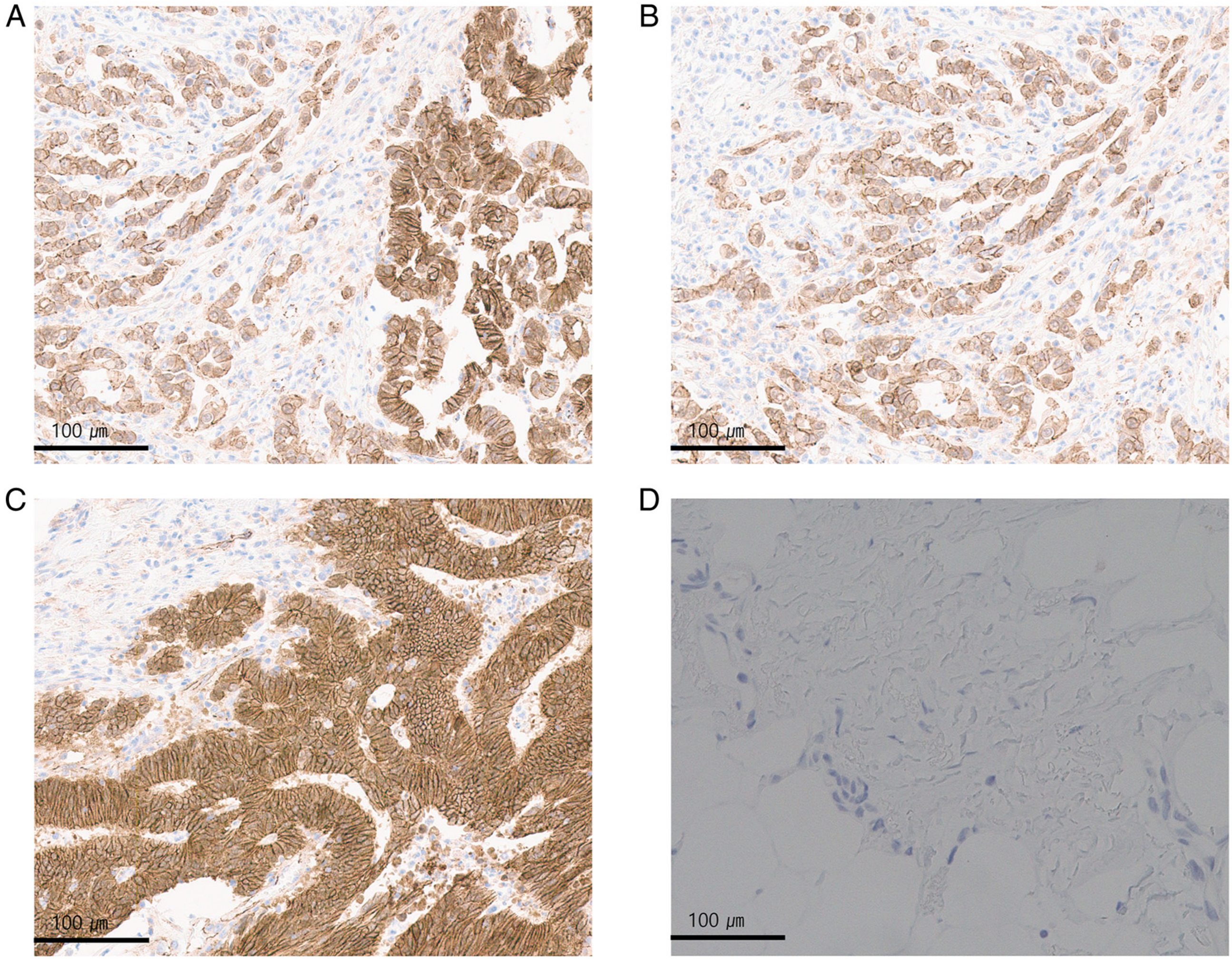

Supplement: Supplementary file 1 [file Supplementary_Data.pdf]
